# A Passive Source Location Method in a Shallow Water Waveguide with a Single Sensor Based on Bayesian Theory

**DOI:** 10.3390/s19061452

**Published:** 2019-03-25

**Authors:** Xiaoman Li, Shengchun Piao, Minghui Zhang, Yan Liu

**Affiliations:** 1 Acoustic Science and Technology Laboratory, Harbin Engineering University, Harbin 150001, China; lixiaoman@hrbeu.edu.cn (X.L.); zhangminghui@hrbeu.edu.cn (M.Z.); liuyan4996@hrbeu.edu.cn (Y.L.); 2 Key Laboratory of Marine Information Acquisition and Security, Harbin Engineering University, Ministry of Industry and Information Technology, Harbin 150001, China; 3 College of Underwater Acoustic Engineering, Harbin Engineering University, Harbin 150001, China

**Keywords:** single sensor, shallow water waveguide, passive location, warping transformation, Bayesian methodology

## Abstract

Bayesian methodology is a good way to infer unknown parameters in a marine environment. A passive source location method in a shallow water waveguide with a single sensor based on Bayesian theory is presented in this paper. The input of a Bayesian inversion algorithm is received different normal mode impulse signals, which are separated and extracted with a warping transformation from received broadband impulse signals. The source range, depth, and other seabed parameters were estimated without prior knowledge of the seabed information. Different normal mode impulse acoustic signals travelling at different group speeds arrived at the sensor at different times because of the dispersion characteristics of the shallow water waveguide. The time delay of different modes can be used for the passive source location. However, normal mode group speeds are greatly affected by the environmental parameters. The performance of the passive location becomes negative when parameters mismatch. In this paper, the source location was transformed to the inversion of the source location and environmental parameters, which can be estimated accurately based on the multi-dimensional posterior probability density (PPD). This method is less limited by environmental factors, and the accuracy of inversion results can be analyzed according to the PPD of inversion parameters, which has higher reliability and a wider application scope. The effectiveness and robustness of the algorithm were quantified in terms of the root mean squared error (RMSE) at a variety of signal-to-noise ratios (SNRs) in 50 simulation sets. The RMSE values decreased with the SNR. The validity and accuracy of the method were proved by the results of simulation and experiment data.

## 1. Introduction

The passive location of underwater sources is the basis of underwater target detection and is an active field in underwater acoustic research. Based on the number of sensors, passive source location technology in a shallow water waveguide can be divided into two categories: methods using a sensor array and those with a single sensor.

For methods with a sensor array (vertical array or horizontal array), the water column is covered by sensors, and the marine environment information can be measured after processing the received signal data. The source will be estimated accurately using the marine environment information. However, in practical applications, a sensor array carries a high computational cost; additionally, the accuracy of the measured data can be greatly affected by the array form, which is influenced by ocean currents and storms [1]. Therefore, passive source methods based on a single sensor have been proposed. These methods sacrifice the environmental information provided by an array but greatly reduce the cost and complexity of recording systems.

Less information is received by a single sensor compared with a sensor array. To solve this issue, passive range methods based on a waveguide invariant and an array invariant are proposed [2,3]. The interference structure characteristics and the relationship between the sound field, distance, and frequency are used in the passive source range method based on the waveguide invariant, but the stability of the interference structure varies with the distance, so the range accuracy depends on the propagation distance [2]. The method based on an array invariant is realized by taking advantage of frequency dispersion, though the range accuracy is also limited to the propagation range [3].

According to normal mode theory [4], the sound source propagation is influenced by environmental parameters in the shallow water waveguide; the received pressure field signal has dispersion and multipath characteristics and is the sum of several normal mode signals. Each number signal contains a significant amount of environmental information, so a single normal mode signal can also be used for passive source location and parameter inversion after analysis and processing. The received signal has multiple components, and each component is a non-linear frequency modulation. To separate and extract the normal mode signals from the received signal, one solution is to transform the received signal so that it adapts to the resolution of the time-frequency (TF) domain, and this can be done using a warping transformation, a model-based transformation designed to linearize the signal phase. Each mode becomes a single-frequency signal with its invariant frequency after the warping transformation [5].

The warping transformation was first used for the Pekeris shallow water waveguide [6]. It was then improved based on beam-displacement ray-mode (BDRM) theory, and the eigen-frequency was closer to the cut-off frequency after the transformation [7]. The warping transformation is constantly being perfected and can be used in a non-ideal shallow water waveguide [8,9] and a range-dependent shallow water waveguide [10]. The warping transformation is now widely used for the passive location of an underwater source [11,12,13,14,15,16,17,18]. For instance, according to the invariant frequency characteristics of the Fourier transform spectrum of the received signal, the approximate relation between the extracted value of the characteristic frequency and the invariant frequency is deduced when the propagation distance is unknown and the source range is estimated by a single sensor [19]. Another robust location method based on the auto-correlation function for a wide-band signal of a single sensor has been presented; a weighting function is constructed to change the peak cross-interference by designing neighboring location constraints. This method tolerates environment mismatch [20]. Source ranges are estimated based on frequency band decomposition and distance weighting when a guided source is employed to provide the crucial frequency invariant features, and the frequency band decomposition is obtained by union processing of autocorrelation function warping spectra of both pressure and particle horizontal velocity. This method can effectively reduce the main lobe width and significantly improve the resolution of source range estimation [21].

The most passive source location methods based on a single sensor require knowledge of the environmental information first. However, it is difficult to obtain detailed and accurate marine information, especially seabed parameters. The Bayesian methodology is a good inversion method with a rigorous evaluation of data errors and model parameterization, which can realize geoacoustic inversion with estimated parameter uncertainties [5]. For the Bayesian inversion method, the inversion scheme can be completed when the marine environmental parameters and sound source are regarded as unknown quantities, so the method has good environmental tolerance [22]. This paper develops and applies the warping transformation and Bayesian theory to estimate the range and depth of the source with seabed parameter uncertainties. The warping transformation is used to extract the normal mode signals, which are used as the input of the Bayesian inversion scheme. The source and power spectral density of the data errors can be estimated by maximizing the likelihood. An optimization algorithm called the genetic algorithm (GA) is used to search the optimum solution [23], and the source range and depth are then inverted. The method is applied to measured data collected in a shallow water experiment in 2014, and the results compare well with global positioning system (GPS) measurements taken during the experiment.

The remainder of this paper is organized as follows: Section 2 describes the separation process for the received signal and the extraction of normal mode signals. Section 3 presents the location theory and algorithms, while Section 4 applies the location method to the simulated data. Section 5 presents and discusses the location results of the experimental data. Finally, Section 6 provides concluding remarks.

## 2. Separation and Extraction of Normal Mode Signals

### 2.1. Modal Propagation Theory

In normal mode theory, the received pressure field signal is the sum of several normal mode signals. Assuming a broadband source emitting at depth *z_s_* in a range-independent shallow water waveguide with a half-infinite liquid seabed, the pressure field *Y*(*f*,*r*) received at depth *z_r_* after propagation over a range *r* can be written as [24]

(1) $$Y(f,r)\approx s(f)\sum_{n=1}^{N}\frac{e^{j\frac{\pi }{4}}}{\sqrt{8\pi }\rho (z_{s})}U_{n}(z_{s},f)U_{n}(z_{r},f)\frac{e^{j\xi_{n}(f)r-\beta_{n}(f)r}}{\sqrt{\xi_{n}r}}$$

where *S*(*f*) is the source spectrum, *N* is the number of normal modes, *ξ_n_*(*f*) is the horizontal wavenumber, and *U_n_* is the modal depth function, whose amplitude varies with depth, *β_n_*(*f*) is the attenuation coefficient, which is a very small value and varies with frequency, and *ρ*(*z_s_*) is the density at the source depth *z_s_*, which is the density of water.

### 2.2. Warping Transformation

*Y*(*f*,*r*) consists of several normal mode signals of different numbers, each number signal contains a significant amount of environmental information, so that a single normal mode signal can also be used for passive source location and parameter inversion after analysis and processing.

A signal processing method called warping [25] is used to separate and extract the normal mode signals from the received pressure field signal. The warping transformation is a model-based transformation designed to linearize the signal phase. Each mode will become a single-frequency signal with its invariant frequency following the warping transformation.

For a given signal *y*(*t*), which is the received signal in the time domain, the warping transformation can be written as [21]

(2) $$Wy(t)=\sqrt{\left|\frac{\partial h(t)}{\partial t}\right|}y\left[h(t)\right]$$

where Wy(t) is the warped signal, and *h*(*t*) is the warping operator while $\frac{\partial h(t)}{\partial t}$ is the derivative of *w*(*t*). Warping transformation is reversible; the warped signal can be unwarped using *h*^−1^(*t*), and $\sqrt{\left|\partial h(t)/\partial t\right|}$ only provides the energy conservation between the original and warped signals.

The warped operator can be written as

(3) $$h(t)=\sqrt{t^{2}+t_{r}^{2}}$$

and the unwarped operator is

(4) $$h^{-1}(t)=\sqrt{t^{2}-t_{r}^{2}}$$

where $t_{r}=\frac{r}{c_{0}}$, and $c_{0}$ is the water sound speed. Warping transformation is robust and can be applied to most low frequency shallow water scenarios without detailed knowledge of the environment or precise propagation range [26]. Therefore, $t_{r}$ can be determined empirically without knowing *r* or $c_{0}$. The received signal can be presented in the TF domain using short-term Fourier transformation (STFT) [27]. Warping transformation is then used in the TF domain, the received signal becomes the sum of several number linear single frequency modes, and the correspondent frequency of each mode is the eigen-frequency. The single normal mode signal is extracted by a frequency filter. The signal in the frequency domain can be transformed from the time domain using Fourier transformation. Therefore, when warping transformation is used, the *n* number normal mode pressure field signal at frequency *f* can be written as

(5) $$Y_{n}\left(r,f\right)\approx s(f)\frac{e^{j\frac{\pi }{4}}}{\sqrt{8\pi }\rho (z_{s})}U_{n}(z_{s},f)U_{n}(z_{r},f)\frac{e^{j\xi_{n}(f)r-\beta_{n}(f)r}}{\sqrt{\xi_{n}r}}.$$

**Figure 1:**
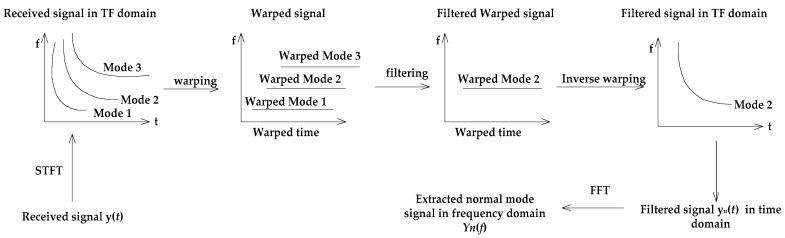
The procedure for the separation and extraction of the normal mode signals using a warping transformation.

The procedure for the separation and extraction of the normal mode signals is described in Figure 1.

## 3. Source Location Scheme

### 3.1. Bayesian Inversion Theory

In a Bayesian inversion, the multi-dimensional posterior probability density (PPD) is usually interpreted in terms of model-parameter estimates and uncertainties. In a Bayesian approach, let **m** be a vector of *M* free parameters representing a realization, and let **d** represent *N* measured data which constrain the model. These quantities are considered random variables that are related via Bayes’ rule [28]:

(6) P(m|d)=P(d|m)P(m)/P(d).

The *P*(**m**|**d**) is the PPD, *P*(**m**) is the prior distribution, *P*(**d**) is the probability density of the measured data, which is independent of **m**, and *P*(**d**|**m**) represents the conditional probability density for **d**, which is interpreted as the likelihood *L*(**m**) for the measured data. Thus, Equation (6) can be written as

(7) P(m|d)∝L(m)P(m)

where

(8) L(m)=P(d|m)∝exp[−E(m)].

The likelihood function depends on the statistical distribution of the data expression and errors (measurement error and theoretical error), and *E*(**m**) is the data misfit function. In cases where the error distribution is not known independently, a good strategy is to choose the Gaussian distribution and estimate the statistical parameters from the data. A generalized misfit combining data and prior can be defined as

(9) φ(m)=E(m)−lnP(m)

where *φ*(**m**) is the cost function. PPD is written as

(10) $$P(m|d)=\frac{\exp[-\varphi (m)]}{\int_{}\exp[-\varphi (m^{\prime },d)]dm^{\prime }}.$$

The integration domain spans an *M*-dimensional parameter space.

In Bayesian inversion, the PPD of **m** is interpreted as the inversion results. In this paper, the maximum a posteriori (MAP) model is used, and the expression is

(11) $$\hat{m}=Arg_{\max}\{P(m|d)\}.$$

Based on Equation (9), Equation (11) can be written as

(12) $$\hat{m}=Arg_{\max}\{L(m)\}=Arg_{\min}\{\varphi (m)\}.$$

Additionally, the mean model, the posterior model covariance matrix, and the one- and two-dimensional marginal probability densities are defined respectively as

(13) $$E(m)=\int m^{\prime }P(m^{\prime }|d)dm^{\prime }$$

(14) $$C_{m}=\int (m^{\prime }-E(m^{\prime })){\left(m^{\prime }-E(m^{\prime })\right)}^{T}P(m^{\prime }|d)dm^{\prime }$$

(15) $$P(m_{i},m_{j}|d)=\int \delta (m_{i}-m_{i}{}^{\prime })\delta (m_{j}-m_{j}{}^{\prime })P(m^{\prime }|d)dm^{\prime }$$

(16) $$P(m_{i}|d)=\int \delta (m_{i}-m_{i}{}^{\prime })P(m^{\prime }|d)dm^{\prime }$$

where ${\left(m^{\prime }-E(m^{\prime })\right)}^{T}$ is the transpose of $\left(m^{\prime }-E(m^{\prime })\right)$.

### 3.2. Cost Function

To estimate the marine environment parameters using Bayesian inversion methodology, a sufficient cost function is necessary.

The traditional Bayesian inversion method is usually carried out by a sensor array (horizontal array or vertical array). Not only can the accuracy of measured data be affected by the array form, the array also carries a significant computational cost when used. In this paper, the different number normal mode signals in frequency are the input of Bayesian methodology, and estimation of the source range and depth is carried out by matching different amounts of normal mode signals in the frequency domain.

Consider the measured data **P**^m^, which is a matrix of *N* × *N_f_*, where *N* is the number of modes, and *N_f_* is the number of frequency points. The element of **P**^m^ at line *n* and column *n_f_* is therefore $p_{nn_{f}}^{m}(m,f)$.

(17) $$p_{nn_{f}}^{m}(m,f)=Y_{n}\left(r,f\right)\approx s(f)\frac{e^{j\frac{\pi }{4}}}{\sqrt{8\pi }\rho (z_{s})}U_{n}(z_{s},f)U_{n}(z_{r},f)\frac{e^{j\xi_{n}(f)r-\beta_{n}(f)r}}{\sqrt{\xi_{n}r}}$$

where $\sum_{n=1}n=N$ and $\sum_{n_{f}=1}n_{f}=N_{f}$. Assuming the data errors are Gaussian-distributed random variables and covariance matrix $C_{nf}^{}$, the likelihood function is

(18) $$L(m,r)=\prod_{n_{f}=1}^{N_{f}}\frac{1}{\pi^{-N}{\left|C_{nf}\right|}^{1/2}}\exp\{-{\left(P_{n_{f}}^{m}-P_{n_{f}}^{c}\right)}^{T}C_{nf}^{-1}(P_{n_{f}}^{m}-P_{n_{f}}^{c})\}$$

where $P_{n_{f}}^{m}$ represents the measured data at the *n_f_*-th frequency point of **P**^c^, $P_{n_{f}}^{c}$ represents the modeled data at the *n_f_*-th frequency point of **P**^c^, and **P**^c^ can be written as

(19) $$P^{c}(m)=H(m)S$$

where **H**(**m**) is the channel transition function, and **S** is the source spectrogram.

In many cases, the error statistics are unknown; the covariance matrices $C_{nf}^{}$ should be estimated from data. Consider first the common approximation of the independent, identical errors and diagonal covariance matrices $C_{nf}^{}=\upsilon_{n_{f}}^{2}I$, where $\upsilon_{n_{f}}^{2}$ is the variance for the *n_f_*-th frequency point and **I** is the identity matrix. The likelihood function is

(20) $$\begin{array}{ll} L(m,r) & =\prod_{n_{f}=1}^{N_{f}}\frac{1}{{\left(\pi \upsilon_{n_{f}}^{2}\right)}^{-N}}\exp\{-{\left|P_{n_{f}}^{m}-P_{n_{f}}^{c}\right|}^{2}/\upsilon_{n_{f}}^{2}\}\\ & =\prod_{n_{f}=1}^{N_{f}}\frac{1}{{\left(\pi \upsilon_{n_{f}}^{2}\right)}^{-N}}\exp\{-{\left|P_{n_{f}}^{m}-H_{n_{f}}^{}S_{n_{f}}^{}\right|}^{2}/\upsilon_{n_{f}}^{2}\} \end{array}$$

where **S***_nf_* is the source spectrum for the *n_f_*-th frequency point. The likelihood function *L*(**m**) can be expressed as the experiential index of the cost function *φ*(**m**), so the likelihood function is

(21) L(m,r)=exp{−φ(m)}.

The cost function is

(22) $$\varphi (m)=\sum_{n_{f}=1}^{N_{f}}\left[N\ln(\pi \upsilon_{n_{f}}^{2})+{\left|P_{n_{f}}^{m}-H_{n_{f}}^{}S_{n_{f}}^{}\right|}^{2}/\upsilon_{n_{f}}^{2}\right].$$

The $\upsilon_{nf}$ and S can be estimated by maximizing the likelihood [29], setting

(23) $$\left\{ \begin{array}{l} \frac{\partial \varphi }{\partial S_{nf}}=0\\ \frac{\partial \varphi }{\partial \upsilon_{n_{f}}^{}}=0 \end{array}\right..$$

When $\frac{\partial \varphi }{\partial S_{nf}}=0$,

(24) $$P_{n_{f}}^{m}=H_{n_{f}}^{}S_{n_{f}}^{}$$

and Equation (24) can be written as

(25) $$H_{n_{f}}^{T}P_{n_{f}}^{m}=H_{n_{f}}^{T}H_{n_{f}}^{}S_{n_{f}}^{}$$

where $H_{n_{f}}^{T}$ is the conjugate transpose matrix of $H_{n_{f}}^{}$, and the estimation of the source spectrum is

(26) $${\tilde{S}}_{n_{f}}^{}={\left(H_{n_{f}}^{T}H_{n_{f}}^{}\right)}^{-1}H_{n_{f}}^{T}P_{n_{f}}^{m}$$

where ${\left(H_{n_{f}}^{T}H_{n_{f}}^{}\right)}^{-1}$ is the inverse matrix of $\left(H_{n_{f}}^{T}H_{n_{f}}^{}\right)$. The ${\tilde{S}}_{n_{f}}^{}$ is substituted for $S_{n_{f}}^{}$ in Equation (20), and the cost function can be written as

(27) $$\varphi (m)=\sum_{n_{f}=1}^{N_{f}}\left[N\ln(\pi \upsilon_{n_{f}}^{2})+{\left|(I-H_{n_{f}}^{}{\left(H_{n_{f}}^{T}H_{n_{f}}^{}\right)}^{-1}H_{n_{f}}^{T})P_{n_{f}}^{m}\right|}^{2}/\upsilon_{n_{f}}^{2}\right].$$

According to $\frac{\partial \varphi }{\partial \upsilon_{n_{f}}^{}}=0$, the estimation of $\upsilon_{n_{f}}^{2}$ is

(28) $$\upsilon_{n_{f}}^{2}=\frac{1}{N}{\left|(I-H_{n_{f}}^{}{\left(H_{n_{f}}^{T}H_{n_{f}}^{}\right)}^{-1}H_{n_{f}}^{T})P_{n_{f}}^{m}\right|}^{2}.$$

Therefore, the cost function is

(29) $$\varphi (m)=\sum_{n_{f}=1}^{N_{f}}\left[2N\ln\left|(I-H_{n_{f}}^{}{\left(H_{n_{f}}^{T}H_{n_{f}}^{}\right)}^{-1}H_{n_{f}}^{T})P_{n_{f}}^{m}\right|+(N+N\ln\pi -N\ln N)\right].$$

When the constant term is ignored, the cost function is

(30) $$\varphi (m)=\sum_{n_{f}=1}^{N_{f}}\left[2N\ln\left|(I-H_{n_{f}}^{}{\left(H_{n_{f}}^{T}H_{n_{f}}^{}\right)}^{-1}H_{n_{f}}^{T})P_{n_{f}}^{m}\right|\right].$$

The source range, depth, and other seabed parameters can be inverted by minimizing *φ*(**m**). The GA is used to search for the optimum solution, when the optimal value of search does not change, and converges to a fixed value, which is considered the optimal value. The mutation probability, selection probability, crossover probability, generation number, and initial population size are 0.05, 0.5, 0.8, 5000, and 64, respectively. In addition, 20 sets are computed in parallel to ensure the parameters converge to the global optimum.

## 4. Simulation Example

The simulation was performed in a shallow water waveguide with a half-infinite liquid seabed. The depth was 25 m, the sound speed in water was an isovelocity with *c*_0_ = 1500 m/s, a broadband source was emitted at depth *z_s_* = 20 m with frequency band 200~300 Hz, the source is a linear frequency modulated impulse signal, the SNR was 20 dB, and the signal was received at depth *z_r_* = 23 m after propagation range *r* = 7700 m. The seabed sound speed was *c_b_* = 1650 m/s, and seabed density was *ρ_b_* = 1.8 g/cm^3^.

### 4.1. The Extraction of Normal Mode

**Figure 2:**
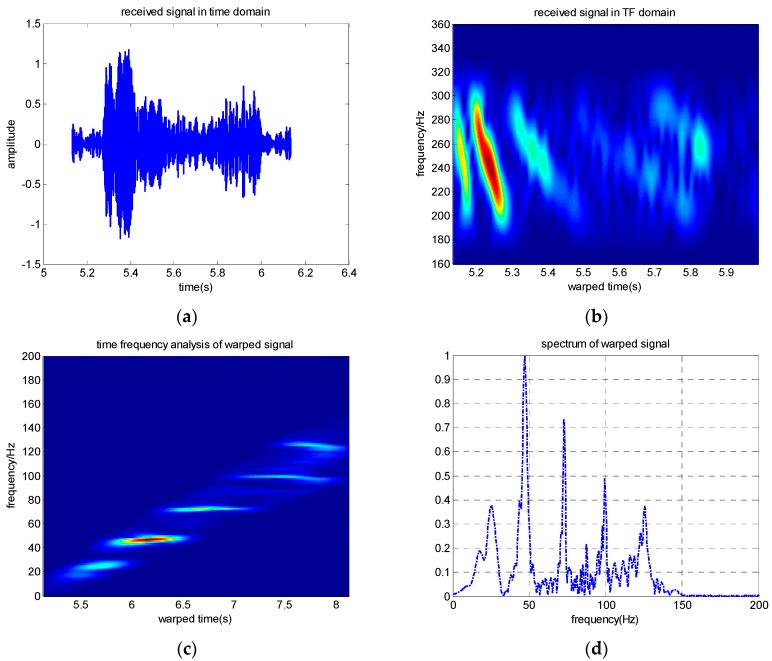
Simulated results. (**a**) Received signal in time domain; (**b**) received signal in the TF domain; (**c**) warped signal in the TF domain; (**d**) spectrogram of the warped signal.

**Figure 3:**
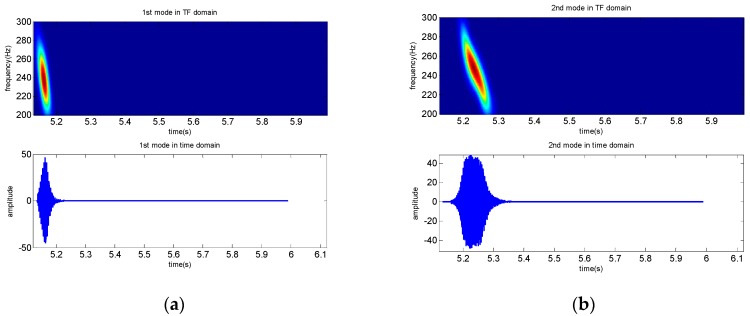

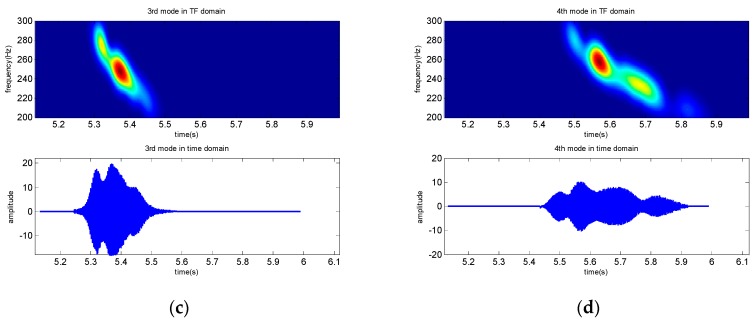
Extracted results of the first four modes, including the mode signal in the TF domain and the time domain, and (**a**–**d**) are results of the 1st to 4th mode, respectively.

**Figure 4:**
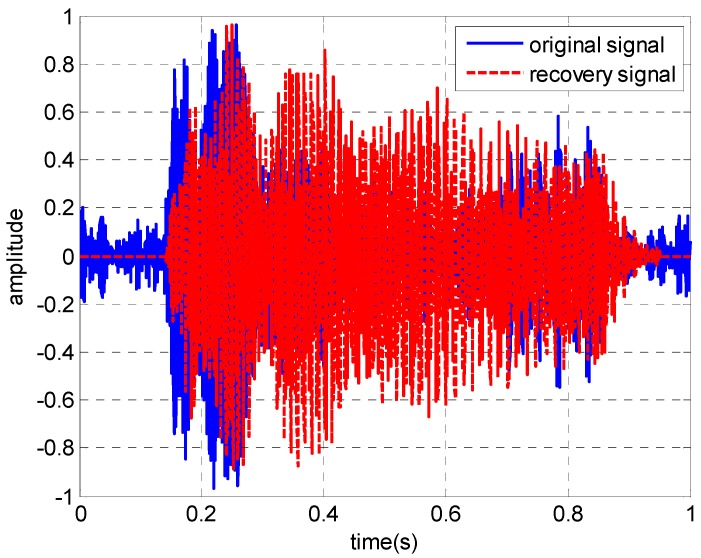
The comparison between the original signal and recovery signal.

The received signal in the time domain and the extracted modes are shown in Figure 2. The received signal contains several normal modes from Figure 2b, Figure 2c shows that the normal mode can be separated after warping transformation, and the phases of the mode signals are transformed from non-linear frequency modulation to linear. Each mode becomes a single-frequency signal with its invariant frequency after warping transformation, and the spectrum characteristics are shown in Figure 2d. Warping transformation is reversible. The mode signals are extracted by a frequency filter and unwarping transformation. The extracted signals in time and TF domains of the first four modes are shown in Figure 3. Additionally, a comparison between the original received signal in the time domain and the signal recovered from the first four warped mode signals was made, and the result illustrates that the recovery signal is consistent with the original signal, but a small amount of noise was ignored (Figure 4).

The input signals of the Bayesian inversion theory are the extracted normal mode signals in the frequency domain. The signal in the frequency domain can be transformed from the time domain using Fourier transformation. When the input signals are obtained, the source depth and range can be estimated based on Section 3.

### 4.2. The Analysis of the Inversion Parameter Sensitivity

**Figure 5:**
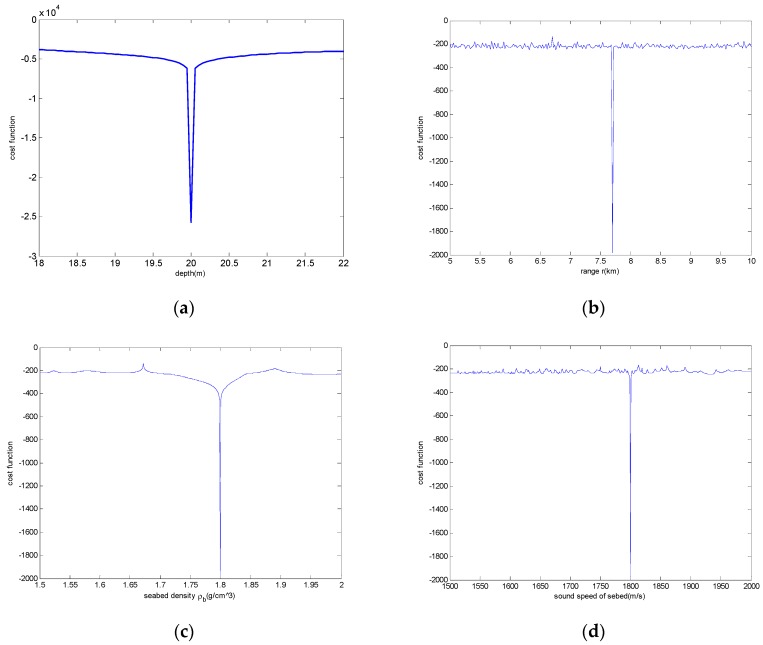
Inversion parameter sensitivity analysis to the cost function. (**a**–**d**) are analysis results of four inversion parameters (source range, depth, seabed sound speed, and seabed density), respectively.

To illustrate the validity of the cost function in Section 3.2, the inversion parameter sensitivity to the cost function is analyzed. When the parameter sensitivity is analyzed, the parameter to be analyzed is constantly changing in a certain range, while the other three parameters remain unchanged. When the analyzed parameter is near the true value, the cost function obtains the minimum value. Four parameters of source range, depth, seabed sound speed, and seabed density are analyzed. The results are as seen in Figure 5; parameters are sensitive to the cost function. The cost function is minimized at the true values, and the analysis curve of parameters varies sharply near the true value, so all inversion parameters are sensitive. The cost function is valid with respect to the inverse parameters.

### 4.3. The Inversion Results

**Figure 6:**
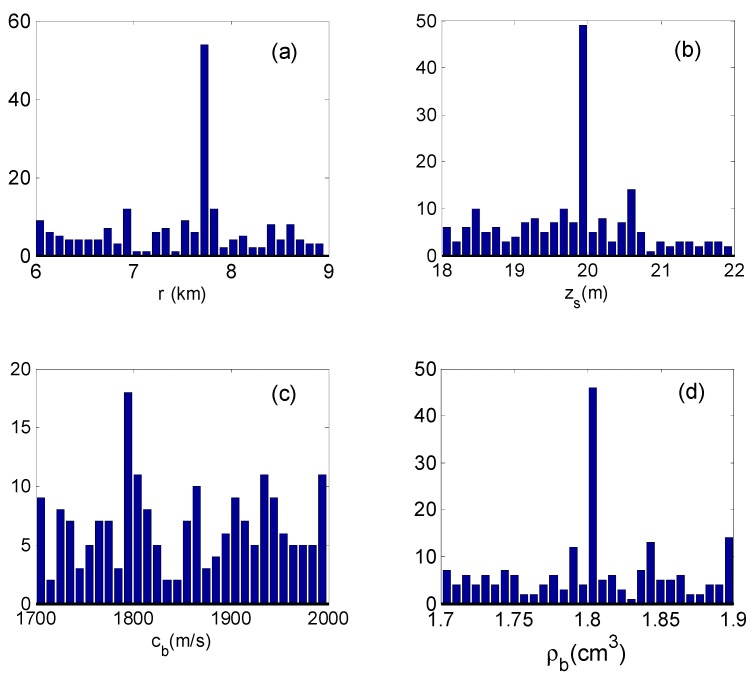
Marginal probability densities from Bayesian inversion: (**a**) the source range; (**b**) the source depth; (**c**) the seabed sound speed; (**d**) the seabed density.

The input signals of the inverse scheme are given in Section 4.1, and the replica (model signal) is computed by KRAKEN, an acoustic computation program [30]. All parameters were searched over relatively wide intervals based on Equation (30) by the GA, and the corresponding search bounds are given in Table 1. When the parameters of the search converge to the optimal value, the optimal value is placed into Equations (10) and (16), and the PPD and the marginal probability densities can be estimated. The inverse value of the parameter corresponds to the maximum marginal probability. Figure 6 presents the marginal probability densities for each individual parameter.

**Figure 7:**
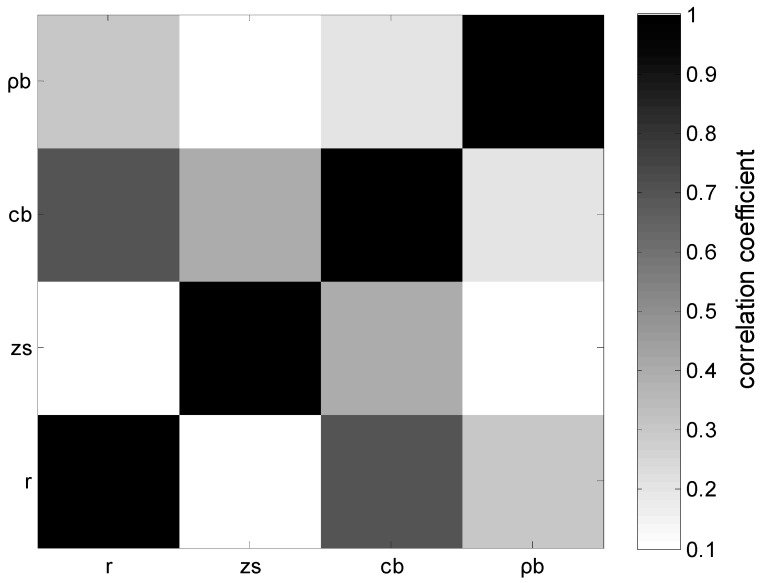
Parameter correlation matrix.

To study the inter-relationship of these parameters, Figure 7 shows the correlation coefficient matrix of arbitrary two inversion parameters calculated by correlating the marginal probability densities of two parameters and normalizing them. Range *r* and source depth *z_s_* correlate with the seabed sound speed ${\tilde{c}}_{b}$, which causes multiple peaks in PPD for *c_b_*. When the inversion parameters have multiple peaks in marginal probability densities, many sets computed in parallel are necessary to ensure that the parameters converge to the global optimum. Other parameters do not indicate a strong correlation.

When there is strong correlation between inverse parameters, the inverse results will be multiply valued. The seabed sound speed ${\tilde{c}}_{b}$ can be estimated by seabed speed empirical formula [29]:

(31) $$c_{b}=2330.4-1257.0\rho_{b}+487.7\rho_{b}^{2}.$$

The inverse parameters were range *r*, source depth *z_s_*, and seabed density *ρ_b_*. The lower the number of inverse parameters, the faster the calculating speed. The multi-valuedness can be avoided, and the location result is more accurate.

The estimated values are the MAP values; these results are listed in Table 1.

According to the estimated results, the source range $\tilde{r}=7.67\;\mathrm{km}$ and ${\tilde{z}}_{s}=19.74\;m$ are close to the true values, and the errors are less than 3%. The estimated values of the seabed parameters are also in a good agreement with the true values. The source range $\tilde{r}$, source depth ${\tilde{z}}_{s}$, and seabed density ${\tilde{\rho }}_{b}$ are well estimated, while the seabed sound speed ${\tilde{c}}_{b}$ is calculated by Equation (31).

### 4.4. The Validity and Robustness of the Algorithm

**Figure 8:**
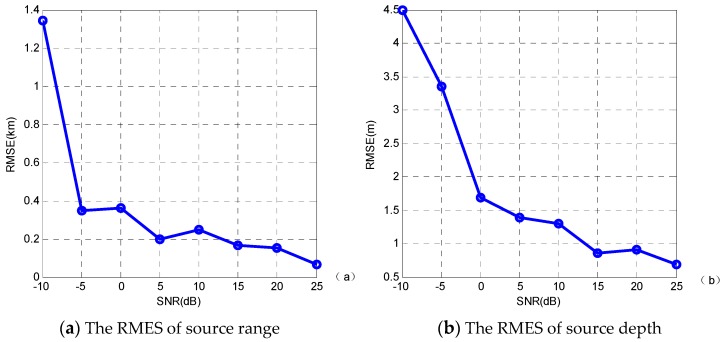
RMSE of different SNRs over the 50 simulations.

To evaluate the effectiveness and robustness of the algorithm, location performance is quantified in terms of the root mean squared error (RMSE) at a variety of SNRs—−10, −5, 0, 5, 10, 15, 20 and 25 dB—in 50 simulation sets. The RMSE can be computed by [31]

(32) $$RMSE=\sqrt{\frac{\sum_{i=1}^{M_{o}}{\left(X_{i}-X_{0}\right)}^{2}}{M_{o}}}$$

where *M_0_* is the number of simulation sets, *X_i_* is the inverse results, and *X_0_* is the true value. In different SNRs, the RMSE of location results (source range and source depth) at a variety of SNRs in 50 simulation sets are shown in Figure 8. Figure 8 shows that the RMSE values decrease with the SNR. When the SNR is higher than 10 dB, the RMSE values near 0. The location results are almost all acceptable.

## 5. Processing Results of Measured Data

**Figure 9:**
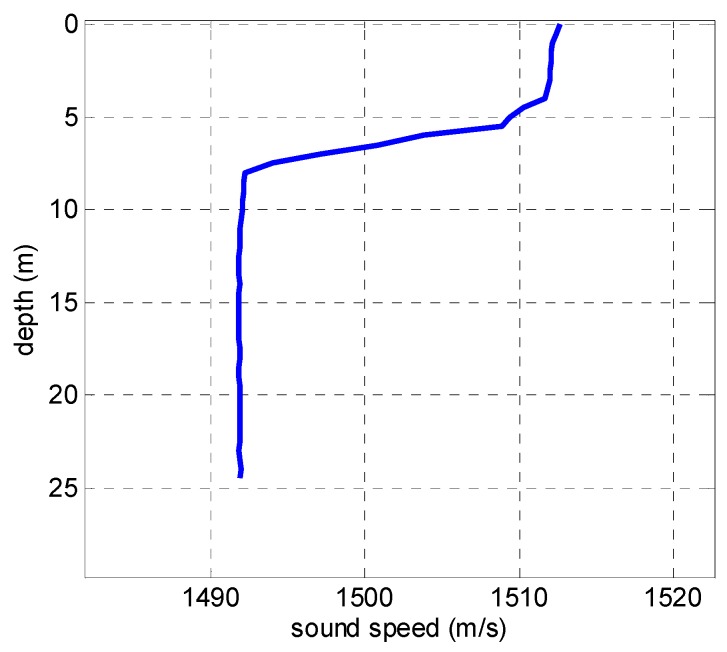
The sound speed profile in water.

**Figure 10:**
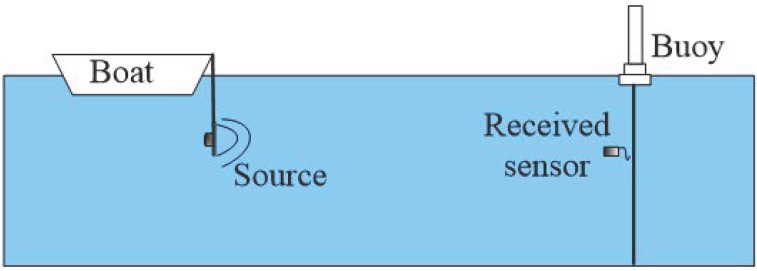
The sound speed profile in water.

The experiment was performed in a shallow water waveguide with a half-infinite liquid seabed in an area of the Yellow Sea, China. The depth is 25 m. The sound speed profile in water is shown in Figure 9. A broadband linear frequency modulated impulse source was emitted by UW350 at depth *z_s_* = 10 m with a frequency band of 200~500 Hz. The signal duration was 3 s, and the signal was received at depth *z_r_* = 9 m by a single sensor after propagation range *r* = 4972 m, which was measured via a global positioning system (GPS). The seabed sound speed *c_b_* and seabed density *ρ_b_* have been inversed by other methods before; the results will here be compared with these inversed results. The equipment of this experiment is shown in Figure 10.

**Figure 11:**
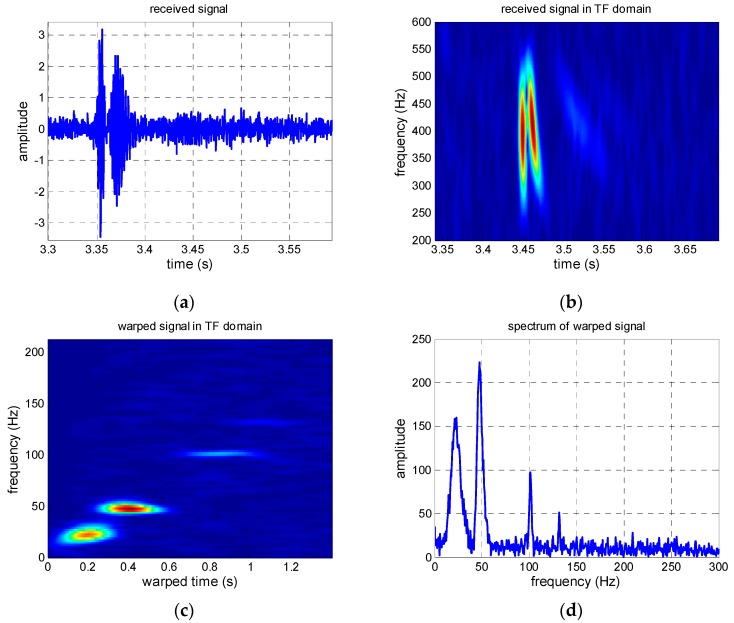
(**a**) Received signal in the time domain; (**b**) received signal in the TF domain; (**c**) warped signal in the TF domain; (**d**) spectrogram of the warped signal.

**Figure 12:**
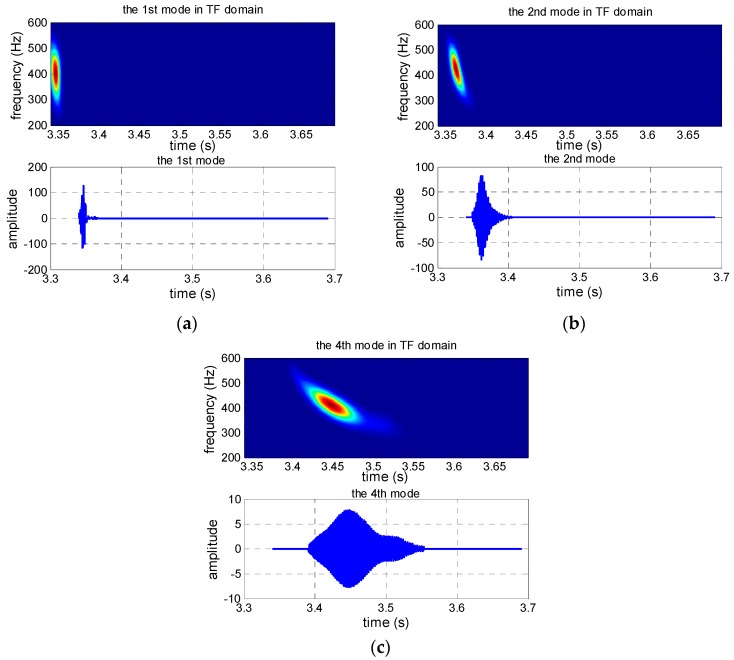
Extracted results of the 1st, 2nd, and 4th modes: (**a**) the 1st mode; (**b**) the 2nd mode; (**c**) the 4th mode.

The pulse compression technique is used to receive signals for convenience. The received signals in the time domain and the TF domain, the warped signal in the TF domain, and the warped signal spectrum are shown in Figure 11a–d, respectively. There are three obvious normal mode signals shown in Figure 11b, while four modes can be seen in Figure 11c,d, which illustrate the advantage of the warping transformation. From Figure 11d, we see that the 3rd mode is faint; one reason is that the received sensor is at the null point of the 3rd mode, and the thermocline in the sound speed profile (SSP) may be another reason. To obtain a better estimated result, the 1st, 2nd, and 4th modes with strong energy were used. The extracted modes are shown in Figure 12.

**Figure 13:**
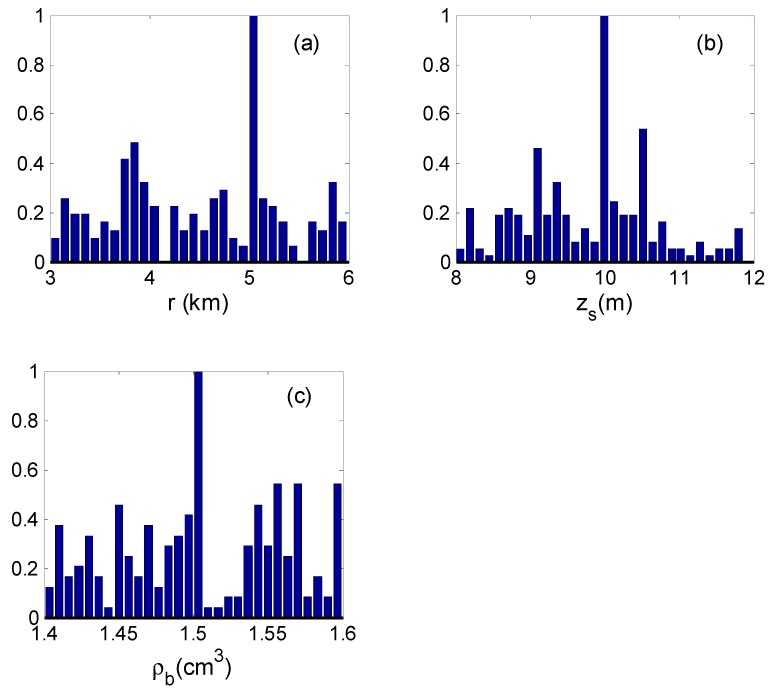
Marginal probability densities from Bayesian inversion: (**a**) the source range; (**b**) the source depth; (**c**) the seabed density.

As in Section 4.3, the inversion results are listed in Table 2, and the marginal probability densities of the inversion parameters are shown in Figure 13.

According to the estimated results, the source range $\tilde{r}$ = 5.04 km was close to the independent range from the GPS data obtained during the experiment (4.792 km), while ${\tilde{z}}_{s}$ = 9.99 m was close to the true value, and the errors of the source range and depth were both less than 10%. The estimated values of the seabed parameters were also in a good agreement with the mean inverted values from other methods. The estimated result was acceptable.

## 6. Conclusions

This paper presents a passive location method of an underwater source based on a single received sensor. Bayesian methodology was used to build the cost function. The methodology was adapted for low frequency propagation in shallow water. In this case, propagation was dispersive, and the received signal consisted of several normal modes, which could be described in the TF domain. A signal processing method called the warping transformation was used to separate and extract different numbers of normal modes, which were then used as the input for the Bayesian inversion scheme. A GA was used to search for the optimum solution, and 10 sets were computed in parallel to ensure the parameters converge to the global optimum.

The key point of this paper is that applying different numbers of normal mode signals instead of array (vertical array or horizontal array) data can reduce cost and avoid measurement errors caused by external environmental factors. Additionally, Bayesian methodology is a good inversion method with a rigorous evaluation of data errors and model parameterization, which can realize geoacoustic inversion with estimated parameter uncertainties, so the source range, depth, and other seabed parameters can be estimated without prior knowledge of the seabed information. The estimated results were in a good agreement with true values and estimated values from other inversion methods.

The inversion parameter sensitivity to the cost function and the inter-relationship of parameters was analyzed. The results illustrate the following: The cost function to every single parameter was effective, and source range r and source depth *zs* correlated with seabed sound speed *c_b_*, while other parameters did not indicate a strong correlation. The seabed sound speed *c_b_* can be estimated by the seabed speed empirical formula to solve the multi-valuedness caused by strong correlations between inverse parameters. The effectiveness and robustness of the algorithm were quantified in terms of the root mean squared error (RMSE) at a variety of signal-to-noise ratios (SNRs) in 50 simulation sets. The RMSE values decrease with the SNR. The method described in this paper was applied to the shallow water waveguide with a half-infinite liquid seabed, and the sound speed in water and the water depth were known, so the application to more complex marine environments can be studied in the future.

## Figures and Tables

**Table 1:** Inversion parameter list. The search bounds, true values, and inversion values are shown.

Inversion Parameter	True Values	Search Bounds	Inversion Values
Range *r* (km)	7.7	[6,9]	7.67
Depth *z_s_* (m)	20	[18,22]	19.74
Seabed sound speed *c_b_* (m/s)	1650	/	1638.168
Seabed density *ρ_b_* (g/cm^3^)	1.8	[1.7,1.9]	1.78

**Table 2:** Inversion parameter list. The search bound, true values, and inversion values are shown. (measured data).

Inversion Parameter	True Values	Search Bounds	Inversion Values in Different Methods			
			This Paper	Ref. [29]	Ref. [32]	Ref. [33]
Range *r* (km)	4.792	[3,6]	5.04	/	/	/
Depth *z_s_* (m)	10	[8,20]	9.85	/	/	/
Seabed sound speed *c_b_* (m/s)	/	/	1615.4	1610.9	1642.1	1590.7
Seabed density *ρ_b_* (g/cm^3^)	/	[1.6,1.8]	1.73	1.71	1.75	1.69
